# Nr1h4 and Thrb ameliorate ER stress and provide protection in the MPTP mouse model of Parkinson’s

**DOI:** 10.26508/lsa.202302416

**Published:** 2024-04-12

**Authors:** Nancy Ahuja, Shalini Gupta, Rashmi Arora, Ella Bhagyaraj, Drishti Tiwari, Sumit Kumar, Pawan Gupta

**Affiliations:** 1 Department of Molecular Immunology, Council of Scientific and Industrial Research, Institute of Microbial Technology , Chandigarh, India; 2 Academy of Scientific and Innovative Research (AcSIR), Ghaziabad, India

## Abstract

Elevated ER stress has been linked to the pathogenesis of several disease conditions including neurodegeneration. In this study, we have holistically determined the differential expression of all the nuclear receptors (NRs) in the presence of classical ER stress inducers. Activation of Nr1h4 and Thrb by their cognate ligands (GW4064 and T3) ameliorates the tunicamycin (TM)-induced expression of ER stress genes. A combination of both ligands is effective in mitigating cell death induced by TM. Further exploration of their protective effects in the Parkinson’s disease (PD) model shows that they reduce MPP^+^-induced dissipation of mitochondrial membrane potential and ROS generation in an in vitro PD model in neuronal cells. Furthermore, the generation of an experimental murine PD model reveals that simultaneous treatment of GW4064 and T3 protects mice from ER stress, dopaminergic cell death, and functional deficits in the MPTP mouse model of PD. Thus, activation of Nr1h4 and Thrb by their respective ligands plays an indispensable role in ER stress amelioration and mounts protective effects in the MPTP mouse model of PD.

## Abstract (precis)

The study highlights the potential of GW4064 and T3 in ameliorating PD as the combination of ligands shows promising effects in reducing ER stress, maintaining mitochondrial activity, and protecting dopaminergic neurons in the MPTP mouse model of PD.

## Introduction

Protein folding majorly takes place in the ER, and any perturbation pertaining to interference with ER homeostasis such as hypoxia, hypoglycemia, protein folding defects, changes in calcium levels, and redox potential causes unfolded and misfolded proteins to accumulate, resulting in a condition called ER stress (Rao & Bredesen, 2004; Kaufman & Malhotra, 2014; Chipurupalli et al, 2019; Kato et al, 2019). Unfolded protein response (UPR) activation is a defense mechanism adopted by our body in response to ER stress, but prolonged or elevated ER stress could switch this defense mechanism to an offensive state, leading to the pathogenesis of various diseases (Minamino et al, 2010; Back & Kaufman, 2012; Cabral-Miranda & Hetz, 2018; Koksal et al, 2021). Critically, high concentrations of misfolded proteins in the ER are sensed by three ER transmembrane proteins, activating transcription factor 6 (ATF6), PERK (protein kinase activated by double-stranded RNA [PKR]-like ER kinase), and IRE1α (inositol-requiring kinase/endoribonuclease 1 α) (Ron & Walter, 2007; Carrara et al, 2013). In unstressed conditions, the three regulators are bound by ER-resident chaperone, binding immunoglobulin protein (BiP) in an inactive state. However, when there is an increase in the load of unfolded proteins, BiP is dissociated from IRE1α, PERK, and ATF6, subsequently leading to their activation (Sommer & Jarosch, 2002; Malhotra & Kaufman, 2007). The initial aim of UPR is to regain homeostasis by activating various genes required for proper protein folding. However, when the levels of misfolded proteins within the cells remain high despite the activation of the pro-survival branch of UPR, the cells initiate terminal UPR, which involves a pro-apoptotic program involving high levels of CAAT/enhancer-binding protein (C/EBP) homologous protein (CHOP)/GADD153 causing cell death (Wang & Ron, 1996). Nuclear receptors (NRs) consist of the superfamily of evolutionarily conserved ligand-activated transcription factors having a key role in various cellular, biological and pathophysiological processes (Francis et al, 2003; Skerrett et al, 2014; Dhiman et al, 2018). NRs regulate complex networks of genes involved in various biological processes. A large body of literature showcases the importance and relevance of NRs in modulating ER stress in diverse biological conditions. Peroxisome proliferator–activated receptor (PPAR)-γ is well known to modulate ER stress, which contributes to its anti-diabetic effects, protects human neural stem cells from amyloid-β–induced neuronal impairment, and prevents the development of mutant huntingtin aggregates in the brain (Chiang et al, 2015; Ikeda et al, 2015; Hong et al, 2018; Lin et al, 2018). Activation of other NRs such as vitamin D receptor (VDR) and liver X receptor (LXR) is also shown to be protective in various disease conditions such as cardiovascular disease, diabetes, and neurodegenerative diseases by modulating ER stress (Riek et al, 2012; Yao et al, 2015; Gavini et al, 2018; Gui et al, 2018; Zhou et al, 2020). The second-most common neurodegenerative condition, Parkinson’s disease (PD), is characterized by the death of dopaminergic neurons in the substantia nigra pars compacta (SNpc) and the presence of misfolded α-synuclein (α-syn) protein in the degenerating neurons forming Lewy bodies (Spillantini et al, 1997; Kalia & Lang, 2015). Dysfunction of various cell organelles such as mitochondria, ER, and Golgi contributes to neuronal loss. It is well established in the literature that NR activation exerts salutary effects in various animal models of PD and has promising preclinical results. Several nuclear receptor–related 1 (Nurr1), LXR, and PPAR agonists exert neuroprotective effects and provide protection in animal models of PD (Dai et al, 2012; Carta, 2013; Kim et al, 2015). Despite the myriad of studies showcasing the importance and relevance of NRs in neurodegenerative diseases, translation of this therapeutic strategy to the clinic is lacking. Treatment of cells with various neurotoxins such as 1-methyl-4-phenylpyridinium (MPP^+^), rotenone, or 6-hydroxydopamine is well-established cellular models of PD, as they mimic pathological disturbances correlated with PD (Hisahara & Shimohama, 2010). These neurotoxins are known to induce several UPR genes triggering ER stress. A plausible mechanism of induction of ER stress by these neurotoxins is the accumulation of damaged oxidized proteins and the generation of reactive oxygen species (ROS) because of the effect of these reagents on mitochondrial respiration (Ren et al, 2021). Moreover, elevated levels of ER stress markers are found in the postmortem brain tissue of PD patients. UPR has been established in several studies to have a critical function in the survival of neuronal cells and hence the pathogenesis of PD. Modulation of UPR by several NRs has been reported in PD. Telmisartan, which is known to activate PPARβ/δ, is shown to be neuroprotective in the rotenone model of PD by attenuating dopamine depletion, reducing the accumulation of α-syn, and relieving ER stress by down-regulating the IRE1α/TRAF2/caspase-12 apoptotic signaling pathway (Tong et al, 2016). Esculetin, an ERR synthetic agonist, alleviated ER stress by reducing the expression of CHOP in primary dopaminergic neurons and is also shown to protect dopaminergic neurons in substantia nigra of 1-methyl-4-phenyl-1,2,3,6-tetrahydropyridine (MPTP) and rotenone-induced PD mouse model (Nakano et al, 2017).

In this study, we have shown the differential expression of all the NRs in the presence of various ER stress inducers and found that two NRs, namely, Nr1h4/farnesoid X receptor and thyroid hormone receptor beta (Thrb), have a similar expression pattern in case of all three ER stressors. We then looked for the effect of activation of these two NRs on ER stress pathway genes using the selective ligand for each NR. We found that treatment with GW4064 and 3,3′,5-triiodo-L-thyronine (T3) relieves ER stress by modulating different branches of UPR. Treatment of neuronal cells with both GW4064 and T3 is much more effective than treatment with either ligand alone in protecting neuronal cells from ER stress–induced cell death. They are equally efficacious in ablating several pathological hallmarks of PD such as ER stress, mitochondrial dysfunction, and ROS production, both in in vitro and in vivo and in human neuronal SH-SY5Y cells. We have shown for the first time that ligand-activated Nr1h4 and Thrb have neuroprotective roles in the MPTP mouse model of PD.

## Results

### NR expression profiling in ER stress–induced differentiated N2a cells

**Figure S1.**
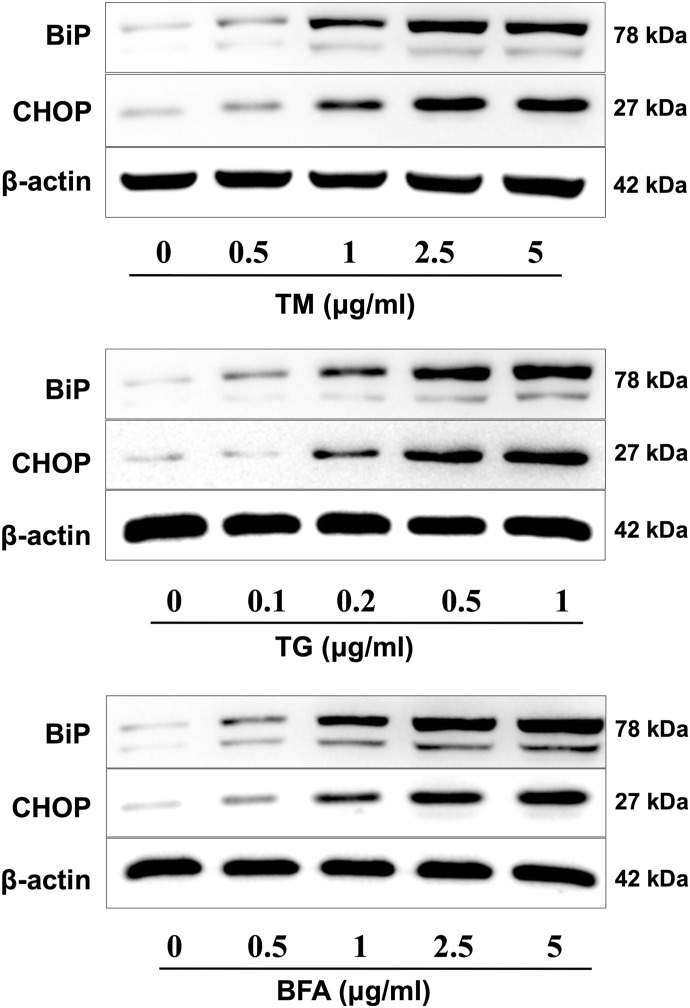
Induction of ER stress in N2a cells. Cells were treated with different concentrations of TM, TG, and BFA, and the expression of ER stress markers such as BiP and CHOP was examined by Western blot. Data are representative of three independent experiments.

**Figure 1.**
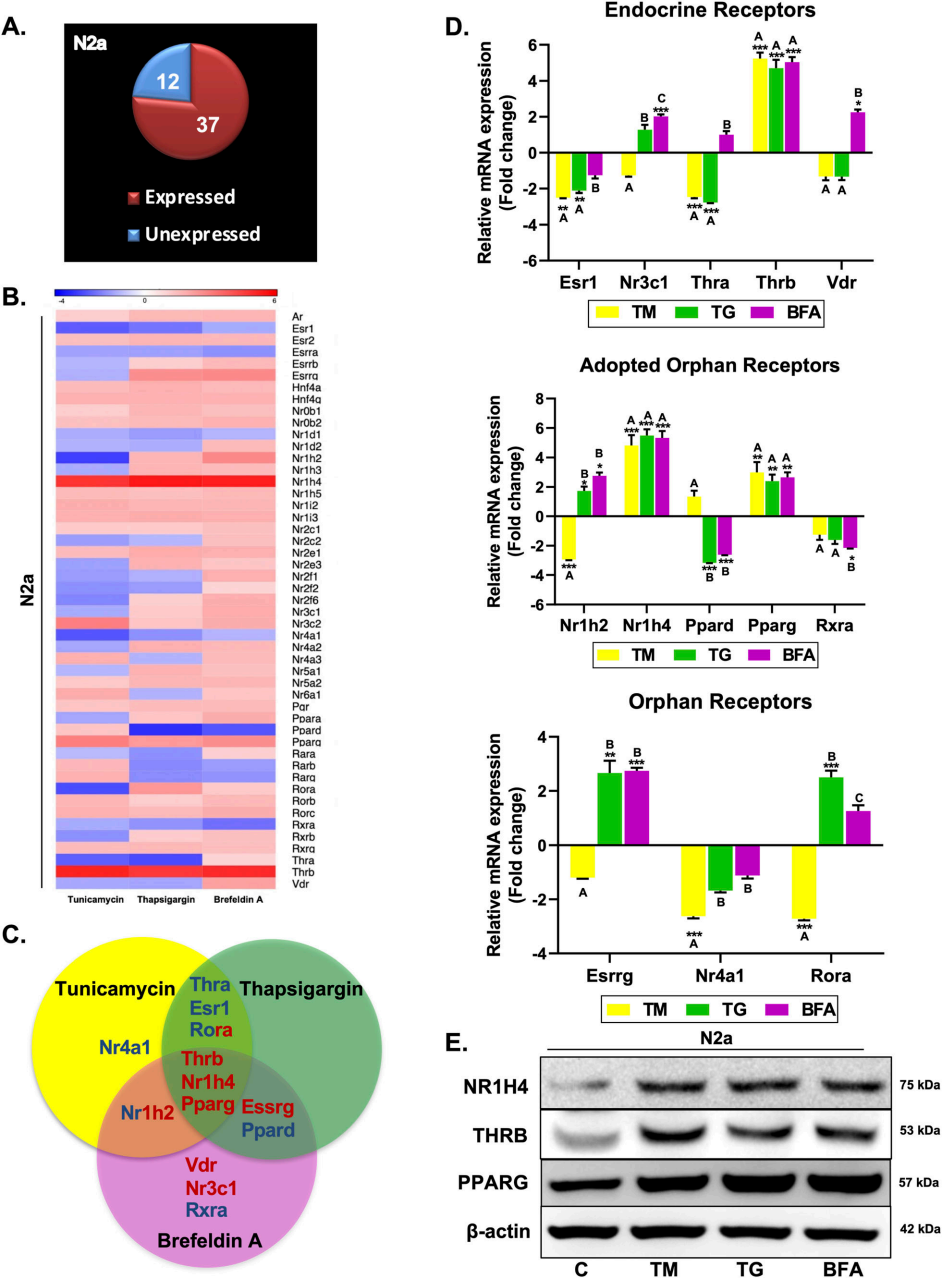
Expression profiling of nuclear receptors in ER stress–induced differentiated N2a cells. Cells were treated with TM, TG, and BFA for 24 h, and the expression of all the NRs was determined by qRT–PCR using an array plate. **(A)** Pie chart depicting the number of NRs that found expression in N2a cells (transcripts having a Ct value 32 or less). **(B)** Heatmap illustrating the altered expression levels of NRs after treatment with TM, TG, and BFA relative to an untreated control. **(C)** Schematic representing the NRs, which are commonly and uniquely modulated (showing at least twofold change) in TM-, TG-, and BFA-induced ER stress conditions. **(D)** Average fold regulation in the expression of endocrine, adopted orphan, and orphan NRs. **(E)** Immunoblot analysis of NRs, which are commonly modulated in all three stress conditions. Data are the mean ± SD or representative of three independent experiments performed in triplicates. *P < 0.05, **P < 0.01, ***P < 0.001 versus untreated control group by a two-tailed *t* test. The letters above the bar depict connecting letter reports representing the correlation of NR expression in the presence of TM, TG, and BFA. Bars not connected by similar letters are significantly different.

Owing to the relevance of NRs in the modulation of ER stress and its subsequent bearing on various disease conditions including neurodegenerative diseases, it is plausible to identify all those NRs having altered expression during ER stress in neuronal cells. To address this, differentiated neuro-2a (N2a) cells were treated with three classic ER stress inducers: thapsigargin (TG, inhibitor of sarcoplasmic/ER Ca2+ ATPase), tunicamycin (TM, inhibitor of N-linked glycosylation), and brefeldin A (BFA, perturbs ER–Golgi protein trafficking) for 24 h. Standard markers for ER stress were analyzed by Western blotting to examine the effect of ER stress inducers on mouse neuroblastoma cells. There was a significant increase in BiP and CHOP at 2.5 μg/ml TM, 0.5 μg/ml TG, and 2.5 μg/ml BFA (Fig S1). Accordingly, these concentrations were selected to induce ER stress and further identification of NR expression using a PCR array. We found that of 49 murine NRs, 37 found expression in differentiated N2a cells (Fig 1A). A complete analysis of all NRs showed that the expression levels of some of the NRs were altered upon induction of ER stress, which is depicted in the heatmap in Fig 1B. 13 NRs were having more than twofold change in their expression pattern as compared to untreated controls, and they are being uniquely or commonly modulated in TM-, TG-, and BFA-induced ER stress conditions (Fig 1C). *Thrb*, *Nr1h4*, and *Pparg* were commonly modulated in all three stress conditions. The expression of endocrine receptor thyroid hormone receptor-α (*Thra*) and estrogen receptor-α (*Esr1*) was down-regulated in the presence of TM and TG. Also, up-regulation of estrogen-related receptor-γ (*Esrrg*) and repression of *Ppard* were observed in the presence of TG and BFA. There were some receptors that were having opposite expression patterns in different stress conditions. *Nr1h2/LXRβ* was repressed in TM-treated cells, whereas its expression was increased in BFA-treated cells; on the contrary, RAR-related orphan receptor-α (*Rora*) was down-regulated in the presence of TM with elevated expression in TG-induced stress conditions. Few NRs were uniquely regulated in either of the stress conditions. Among these, *Nr4a1* was only modulated in cells treated with TM, whereas *Vdr*, *Nr3c1/GR* (glucocorticoid receptor), and retinoid X receptor-α (*Rxra*) were modulated in BFA-treated cells (Fig 1D). We then looked for the protein expression of those NRs, which were commonly modulated in all three stress conditions, and in consistent with their mRNA expression, the protein levels of all three NRs were up-regulated (Fig 1E). Collectively, our results ascertained the expression pattern of all the NRs in mouse neuronal cells. Although some of these receptors have already been shown to modulate ER stress including Pparg, Ppard, Nr1h2, Vdr, few NRs were not previously known to have any implications in ER stress–related pathological conditions (Chiang et al, 2015; Toral et al, 2016; Gavini et al, 2018; Zhou et al, 2020). This calls for further detailed studies to decipher their unidentified functions in modulating ER stress biology in neurons.

### Nr1h4 and Thrb activation ameliorates TM-induced ER stress in differentiated N2a cells

Nr1h4 is a bile acid–sensing NR that has a predominant role in the liver where it regulates triglyceride levels, cholesterol, bile acids, and glucose metabolism (Panzitt & Wagner, 2021). However, recent studies have shown its additional functional roles in different tissues and several disease conditions. There are few reports where activation of Nr1h4 has been shown to alleviate ER stress and hence provide protection in various pathological conditions such as diabetic tubulopathy and non-alcoholic fatty liver disease, and in liver injury caused by ER stress (Gai et al, 2017). Thyroid receptors belong to a class of endocrine receptors having cardinal roles in metabolism, and the development and dysregulation of these receptors has ramifications on human health (Torres-Manzo et al, 2018). Despite the availability of a large body of literature stating the importance of these two receptors in other tissues, little is known about their relevance in the brain. The common modulation, higher fold change, and unrecognized role of Nr1h4 and Thrb in the modulation of ER stress in neuronal cells instigated us to investigate their functional relevance. As the effect of ER stress inducers on the mRNA and protein expression of both the receptors was similar, we chose to use TM for induction of ER stress in all further experiments.

**Figure 2.**
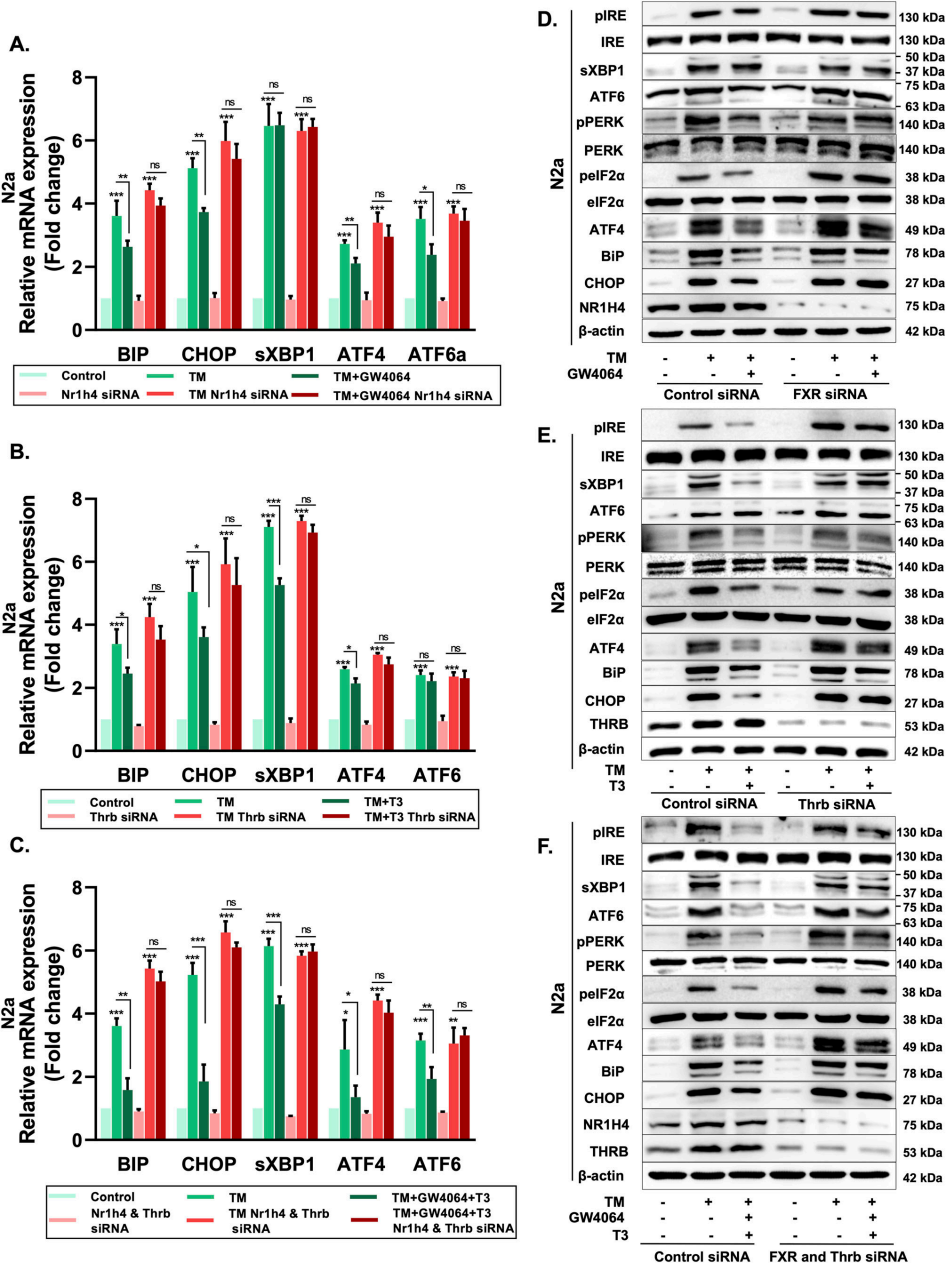
Nr1h4 and Thrb activation ameliorates TM-induced ER stress in differentiated N2a cells. **(A, B, C)** qRT–PCR analysis of ER stress genes in control and (A) Nr1h4 knockdown, (B) Thrb knockdown, or (C) Nr1h4 and Thrb double-knockdown differentiated N2a cells, treated with TM, in the presence or absence of GW4064 or T3 or both. **(D, E, F)** Immunoblot analysis of all the ER stress markers involved in three UPR pathways (IRE, PERK, and ATF6) in control and (D) Nr1h4 knockdown, (E) Thrb knockdown, or (F) Nr1h4 and Thrb double-knockdown differentiated N2a cells, treated with TM, in the presence or absence of GW4064 or T3 or both. Data are the mean ± SD or representative of three independent experiments performed in triplicates. *P <= 0.05, **P < 0.01, ***P < 0.001 versus control group or as indicated by a two-tailed *t* test.

**Figure S2.**
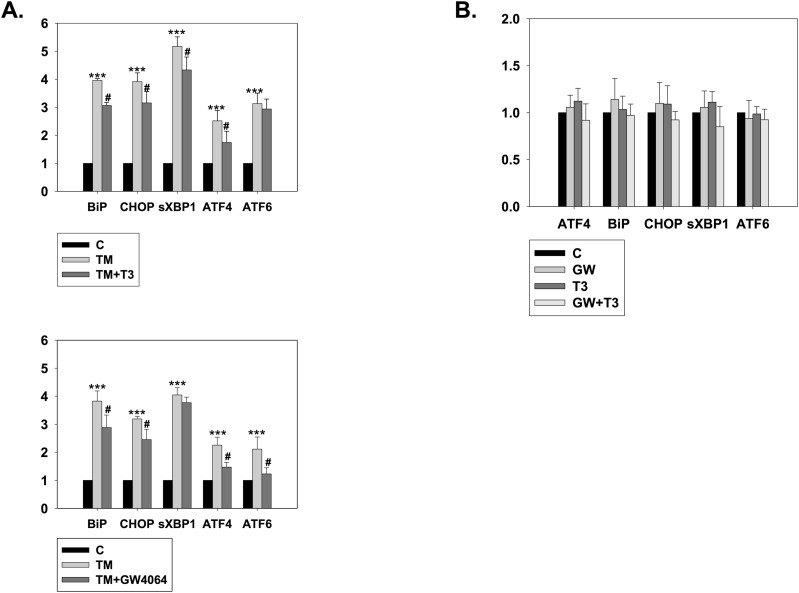
Nr1h4 activation in Thrb knockdown background and Thrb activation in Nr1h4 knockdown background ameliorate TM-induced ER stress. **(A)** qRT–PCR analysis of ER stress genes in (A) Nr1h4 knockdown N2a cells, treated with TM, in the presence or absence of T3; **(B)** Thrb knockdown N2a cells, treated with TM, in the presence or absence of GW4064; and **(C)** N2a cells in the absence or presence of GW4064 or T3 or both. Data are the mean ± SD or representative of three independent experiments performed in triplicates. ***P < 0.001 versus control group; #*P* < 0.05, compared with TM by a two-tailed *t* test.

To characterize their role in the modulation of ER stress, gain-of-function and loss-of-function experiments were done. Control or Nr1h4 knockdown N2a cells were treated with TM, either with or without GW4064, a pharmacological activator of Nr1h4. Similarly, we treated control and Thrb knockdown cells with TM in the presence or absence of T3 (ligand for Thrb). We then looked for the expression of several genes majorly involved in three arms of UPR pathways such as *BiP*, *CHOP*, spliced X-box binding protein 1 (sXBP1), activating transcription factor 4 (*ATF4*), and *ATF6α*. TM treatment increased the expression of all five ER stress response genes, whereas treatment with either GW4064 or T3 abrogated the TM-induced gene expression of *BiP*, *CHOP*, and *ATF4* significantly (Fig 2A and B). GW4064 treatment did not affect the expression of sXBP1, whereas it abrogated TM-induced *ATF6α* expression (Fig 2A). On the contrary, treatment with T3 led to the reduced expression of sXBP1, whereas there was little to no effect on the expression of *ATF6α* (Fig 2B). The absence of either Nr1h4 or Thrb led to the induction of ER stress upon TM treatment, whereas treatment with their cognate ligands (GW4064 or T3) did not rescue the TM-induced stress (Fig 2A and B). Furthermore, to ascertain the individual effect of receptor–ligand pair, we treated Nr1h4 knockdown cells with T3 and Thrb knockdown cells with GW4064 in the presence of TM. There was down-regulation of *BiP*, *CHOP*, and *ATF4* in either case. However, *ATF6α* was only modulated by GW4064, and sXBP1 only by T3 (Fig S2A). To scrutinize the effect of the combination of ligands on the ER stress genes, cells were treated with TM in the presence or absence of GW4064 and T3. We observed that the expression of *BiP*, *CHOP*, sXBP1, *ATF4*, and *ATF6α* was significantly down-regulated in combination treatment as compared to either ligand alone. However, the absence of both receptors failed to repress UPR genes (Fig 2C). We also looked for the expression of all these genes in the presence of individual ligands and in cells treated with the combination of ligands where no significant difference in gene expression was observed (Fig S2B).

To further verify the above results, the effect of activation of these receptors on UPR pathway proteins was investigated. We looked for the protein levels of p-IRE1α, IRE-1α, sXBP1, ATF6α, p-PERK, PERK, phosphor-eukaryotic initiation factor 2 alpha (p-eIF2α), eIF2α, ATF4, BiP, and CHOP in control, Nr1h4 knockdown, Thrb knockdown, and Nr1h4 and Thrb double-knockdown N2a cells. Consistent with mRNA data, similar results were obtained at protein levels (Fig 2D–F). GW4064 was able to ameliorate TM-induced ER stress by suppressing the p-eIF2α/ATF4/CHOP and ATF6α axis of UPR pathways (Fig 2D), whereas T3 is exerting its protective effect by impeding the p-eIF2α/ATF4/CHOP and IRE-1α/XBP1/sXBP1 axis (Fig 2E) in differentiated N2a cells. As a result, the GW4064 and T3 combination was more efficient in mitigating TM-induced ER stress as compared to treatment with either ligand alone (Fig 2F).

### Treatment with Nr1h4 and Thrb agonists prevented differentiated N2a from ER stress–mediated cell death

**Figure 3.**
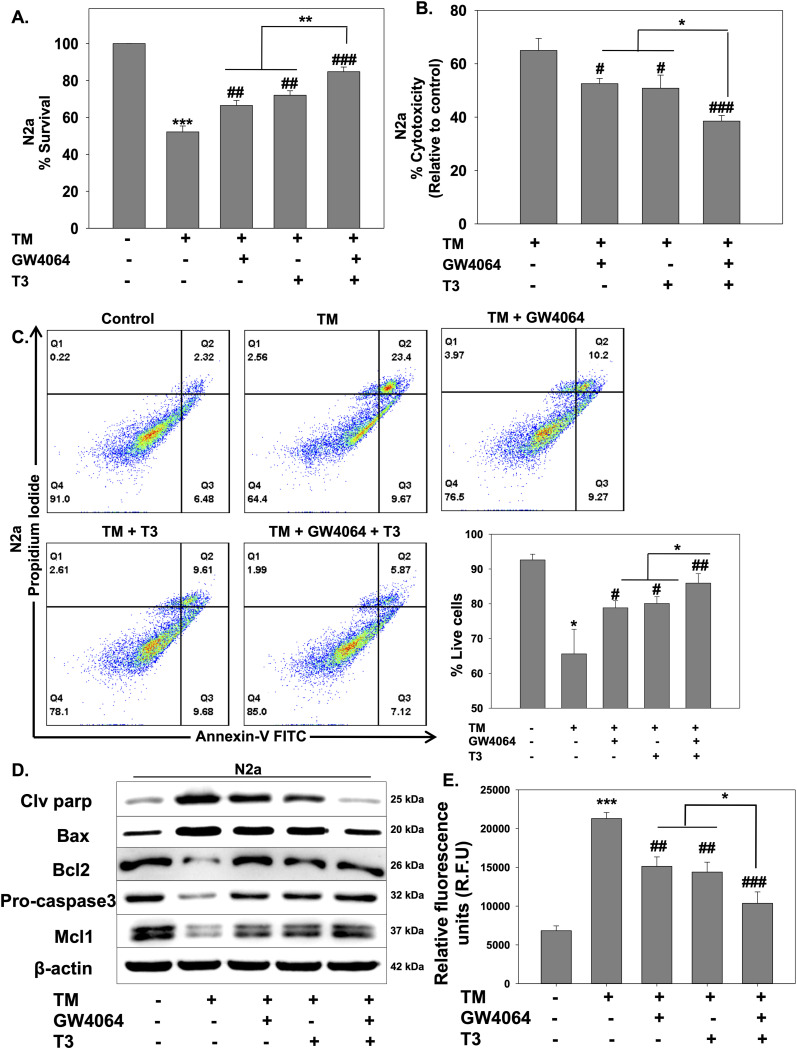
GW4064 and T3 treatment is protective in ER stress–mediated cell death. **(A, B, C)** Cells were treated with TM in the absence or presence of GW4064 or T3 or both for 24 h, and cell death was monitored by a (A) MTT assay, (B) LDH release assay, and (C) annexin-V and PI staining by flow cytometry. **(D)** Immunoblot analysis of pro-apoptotic (clv parp, Bax) and anti-apoptotic (Mcl-1, Bcl-2, pro-caspase-3) markers. **(E)** Cleaved caspase-3 was measured by a fluorescent assay. Data are the mean ± SD or representative of three independent experiments performed in triplicates. *P < 0.05, **P < 0.01, ***P < 0.001, compared with control or as indicated; #*P* < 0.05, ##*P* < 0.01, ###*P* < 0.001, compared with TM by a two-tailed *t* test.

Elevated ER stress leads to cell death (Sano & Reed, 2013). We next sought to look for the effects of the combination of ligands on cell survival and death employing both 3-(4,5-dimethylthiazol-2-yl)-2,5 diphenyltetrazolium bromide (MTT) assay and lactate dehydrogenase (LDH) assay. Treatment with TM leads to reduced cell viability (Fig 3A) and more LDH release (Fig 3B), which were rescued upon the addition of GW4064 or T3. Furthermore, an increase in cell viability (up to 85%) and a decrease in LDH release were noted when the cells were incubated with both ligands, indicating that TM-induced cytotoxicity could be remarkably attenuated by the combination of ligands (Fig 3A and B). To further discern their effects on ER stress–induced apoptosis, cells were treated with TM alone or in the presence of GW4064 or T3 or both for 24 h. Apoptosis was determined by an annexin-V/propidium iodide (PI) assay. Results revealed that the presence of TM significantly elevated the number of apoptotic cells, which was reduced upon individual ligand treatment as detected by flow cytometry analysis, whereas the presence of both the ligands further decreased the number of cells in apoptosis (Fig 3C). Furthermore, the expression of ER stress–related apoptotic proteins such as pro-apoptotic cleaved PARP and Bax and anti-apoptotic Bcl-2, Mcl-1, and pro-caspase-3 was detected by Western blot. We observed that pro-apoptotic protein levels were increased in the presence of TM, which were reduced in the presence of GW4064 or T3, whereas a significant reduction was observed in combination treatment (Fig 3D). Cleaved caspase-3 levels were detected by a fluorescent assay where similar results were obtained (Fig 3E). These findings substantiate the neuroprotective role of Nr1h4 and Thrb in ER stress–induced cell death.

### Nr1h4 and Thrb activation is protective in an in vitro PD model

**Figure 4.**
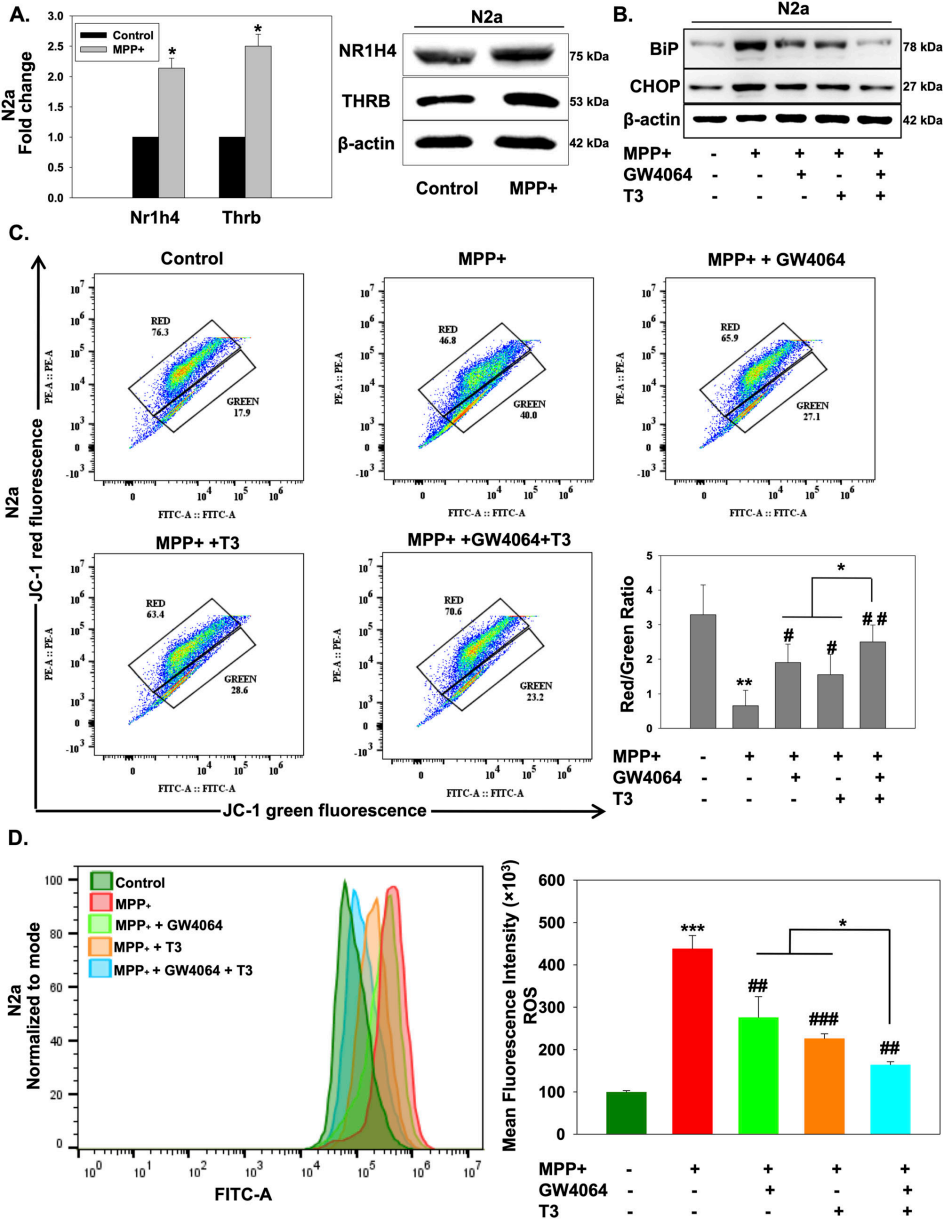
Activation of Nr1h4 and Thrb by GW4064 and T3 prevents mitochondrial membrane depolarization and ROS production in an in vitro PD model. Differentiated N2a cells were treated with MPTP in the absence or presence of GW4064 or T3 or both for 24 h. **(A)** qRT–PCR and immunoblot analysis of Nr1h4 and Thrb in the presence of MPP^+^. **(B)** Immunoblot of ER stress markers BiP and CHOP. **(C)** Mitochondrial membrane potential was measured by flow cytometry using the fluorescent probe JC-1. **(D)** Total ROS levels were detected with the fluorescent probe, 2′,7′-dichlorodihydrofluorescein diacetate, by flow cytometry. Data are the mean ± SD or representative of three independent experiments performed in triplicates. *P < 0.05, **P < 0.01, ***P < 0.001, compared with control or as indicated; #*P* < 0.05, ##*P* < 0.01, ###*P* < 0.001, compared with MPP^+^ by a two-tailed *t* test.

Emerging data suggest a plausible implication of ER stress in the pathogenesis of PD (Mou et al, 2020). MPP^+^, a dopaminergic neuron-specific neurotoxin that causes symptoms of PD, has been frequently used as a model for studying the pathways of cell death in PD (Hutter-Saunders et al, 2012). We first looked for the expression of Nr1h4 and Thrb upon MPP^+^ exposure where we found the increased expression of both these receptors at mRNA and protein levels (Fig 4A). We used a 500 μM MPP^+^ concentration as it is the lowest concentration that decreased the cell viability significantly as observed by us and reported by other groups (Mazzio & Soliman, 2012; Su et al, 2021; Mahmoudian Esfahani et al, 2023).

To elucidate the effect of MPP^+^ treatment on ER stress in differentiated N2a cells, we treated the cells with 500 μM MPP^+^ for 24 h along with either ligand alone or in combination. Ligand treatment attenuated the MPP^+^-induced increase in BiP and CHOP expression, whereas a significant reduction was observed in combination treatment. These results indicate that activation of Nr1h4 and Thrb is abrogating ER stress in an in vitro PD model (Fig 4B).

**Figure S3.**
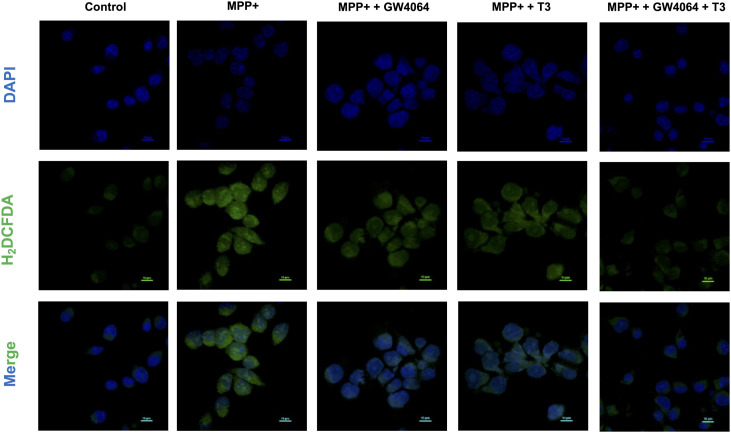
GW4064 and T3 treatment reduced MPP^+^-induced ROS production. Total ROS levels were detected with the fluorescent probe, 2′,7′-dichlorodihydrofluorescein diacetate, by confocal imaging. Data are representative of three independent experiments.

Mitochondrial dysfunction is one of the hallmarks of PD (Helley et al, 2017). To scrutinize the role of mitochondria in MPP^+^-induced neuronal cell death, cells were treated with MPP^+^ to monitor mitochondrial membrane potential (Δψm). MPP^+^ treatment for 24 h caused dissipation of Δψm, as evidenced by a decrease in the red-to-green fluorescence ratio of JC-1, as compared to untreated controls. However, administration of either GW4064 or T3 attenuated MPP^+^-induced Δψm loss as indicated by an increase in fluorescence intensity. Simultaneous administration of both ligands abolished the effect of MPP^+^ on Δψm suggesting their neuroprotective relevance (Fig 4C). Exaggerated generation of ROS has been proposed to be a crucial mechanism underlying the cytotoxicity of MPP^+^ (Dias et al, 2013). We measured total ROS production using the fluorescent dye 2′, 7′-dichlorodihydrofluorescein diacetate (H_2_DCF-DA) by both flow cytometry (Fig 4D) and confocal imaging (Fig S3). ROS levels were markedly augmented in MPP^+^-exposed cells, whereas treatment with either GW4064 or T3 rescued these elevated levels, which were further decreased significantly in GW4064 and T3 combination treatment.

These results demonstrate that the combination of GW4064 and T3 protected mitochondrial function and suppressed MPP^+^-induced ROS production in differentiated N2a cells.

### Co-administration of GW4064 and T3 protected mice from MPTP intoxication

**Figure 5.**
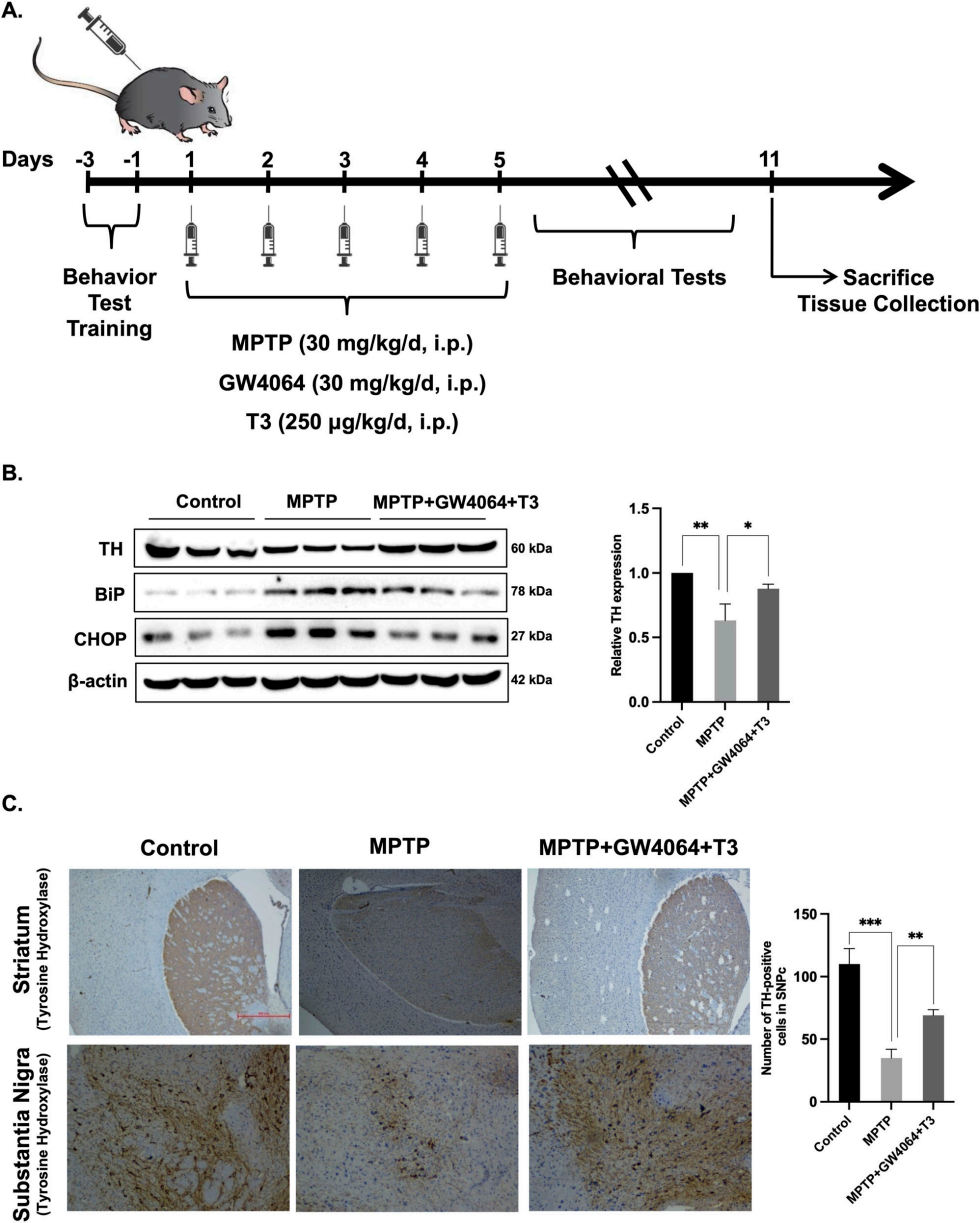
Treatment with GW4064 and T3 has neuroprotective effects in the MPTP mouse model of PD. **(A)** Schematic representation of the MPTP model experimental design. **(B)** Immunoblot of TH and ER stress markers (BiP and CHOP) in the striatum. **(C)** Immunohistochemical detection of TH in the striatum and the SNpc. Scale bars: 50 μm. Data are representative of three independent experiments. *P < 0.05, **P < 0.01, ***P < 0.001, compared with control or as indicated by a two-tailed *t* test.

To validate the in vitro findings, in an in vivo setting, we used the MPTP mouse model of PD. MPTP is extensively employed as a model for investigating the molecular and cellular mechanisms, as well as neuropathological events in PD (Mustapha & Mat Taib, 2021). Animals were divided into three groups: control group, MPTP group, and MPTP along with GW4064 and T3 group. A schematic representation of the MPTP mouse model of the PD experimental design is depicted in Fig 5A. We first measured the protein levels of BiP and CHOP (ER stress markers) in the striatum by immunoblotting, and we found that elevated levels were found in MPTP-treated mice as compared to control mice. BiP and CHOP were significantly reduced in the striatum of ligand-treated mice (Fig 5B). The rate-limiting enzyme tyrosine hydroxylase (TH) is known to produce dopamine in the brain of mice. We measured the amount of TH by Western blot (Fig 5B) and immunohistochemistry (IHC) (Fig 5C). A significant reduction in TH immunoreactivity was observed in the striatum of MPTP-treated animals as compared to the equivalent regions of control animals. Interestingly, there was an increase in TH expression and TH-positive neurons in the SNpc of animals that were subjected to GW4064 and T3 treatment. These data suggest that pretreatment of the combination of GW4064 and T3 in mice is protective not only in curtailing ER stress but also in safeguarding dopaminergic neurons from cell death.

**Figure 6.**
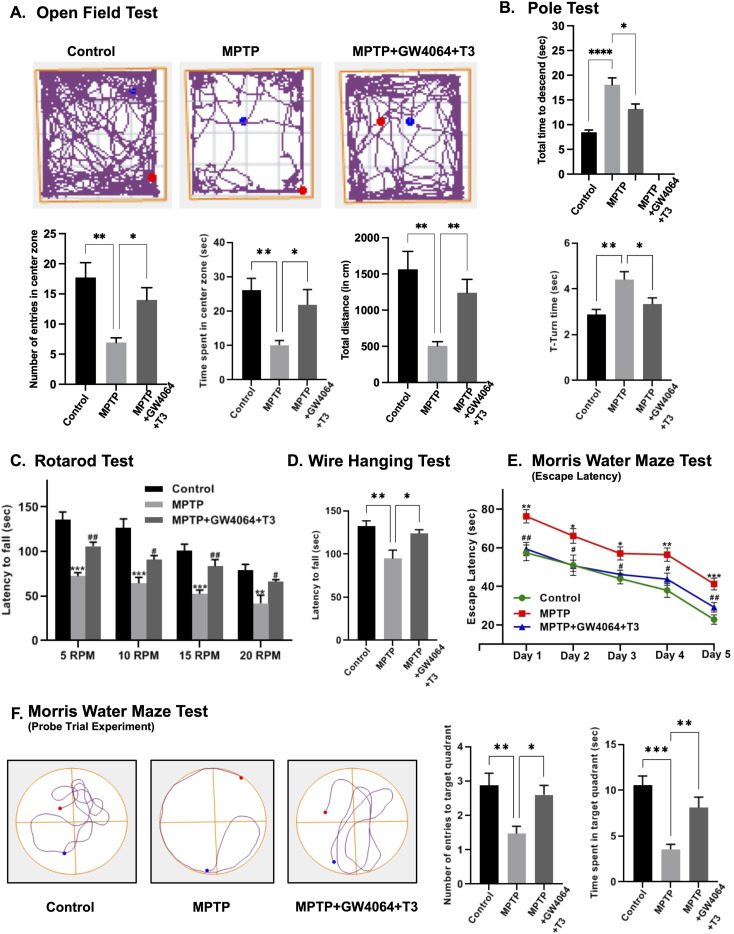
Behavioral analysis of the GW4064- and T3-administered MPTP mouse model of PD. **(A)** Open field test results of MPTP-treated mice. Track plots depicting the movement of mice in the open field test for 15 min using ANY-maze software. The number of entries in the center zone, time spent in the center zone, and total distance traveled in 15 min in an open field. **(B)** Pole test results where the total time to descend and T-turn time were recorded. **(C)** Rotarod test where latency to fall off the rotarod is depicted at different rotation speeds (5–20 rpm). **(D)** Wire hanging test to measure the grip strength. **(E)** Morris water maze tests where mice were analyzed for the escape latency during a 5-d training course. **(F)** In the probe tests, the track plot of the movement of mice for 90 s, the number of entries into the target zone, and the time spent in the target zone are depicted. Data are presented as the mean ± SEM (n = 15/group). *P < 0.05, **P < 0.01, ***P < 0.001, ****P < 0.0001, compared with the control group or as indicated; #*P* < 0.05, ##*P* < 0.01 for MPTP versus MPTP+GW4064+T3 group by one-way ANOVA followed by either the Tukey post hoc analysis (in cases of homogeneous variance) or Dunnett’s T3 test (for heterogeneous variance).

Next, to determine the functional deficits caused by MPTP, we performed various behavioral tests such as open field test (OFT) for general locomotor activity, Morris water maze for cognitive decline, pole test, narrow beam walk, and rotarod for coordination, and an elevated plus maze for determining anxiety-related behavior. In OFT, the movement of mice in the open field was monitored using ANY-maze video tracking software. Various parameters such as the overall distance traveled, the number of entries, and the amount of time spent in the center zone during the 15-min test duration were recorded. MPTP intoxication caused a marked decrease in the number of entries and time spent in the center zone as compared to the control group indicating anxiety-like behavior. Also, MPTP-treated animals were less active and traveled smaller distances compared with animals that did not receive MPTP. Interestingly, GW4064 and T3 significantly improved MPTP-induced anxiety and hypolocomotion (Fig 6A).

**Figure S4.**
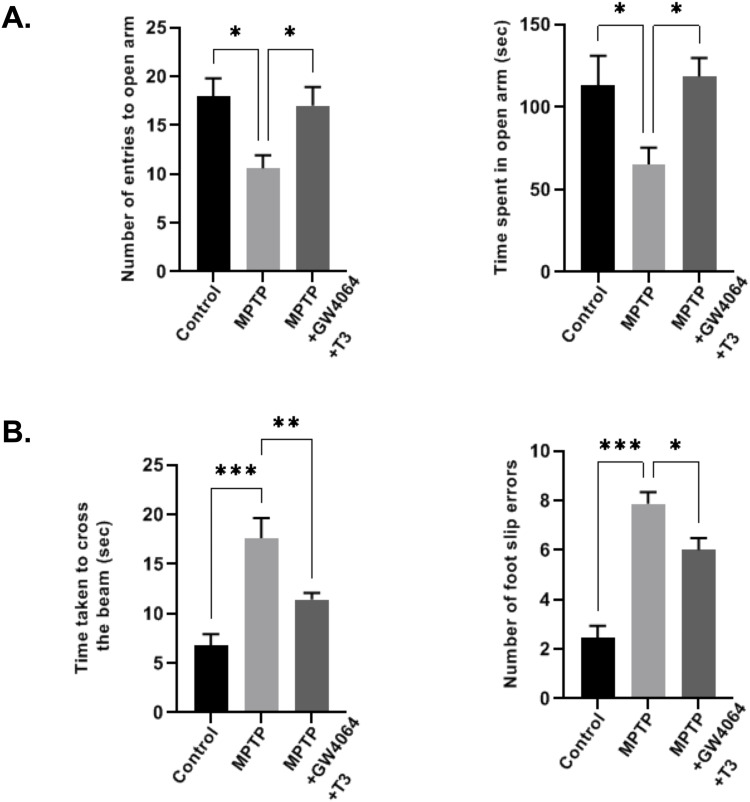
Anxiety and locomotor activity of MPTP-induced PD mouse model. **(A)** EPMT results where the number of entries into and time spent in open arms are depicted. **(B)** Narrow beam walk test results depicting the time taken to cross the beam and the number of foot slip errors. Data are presented as the mean ± SEM (a total of 15 mice per group were used; *P < 0.05, **P < 0.01, ***P < 0.001, compared with the control group or as indicated, by one-way ANOVA followed by the Tukey post hoc test).

Given that non-motor symptoms such as anxiety are frequently reported in PD patients, we performed an elevated plus-maze test (EPMT). MPTP treatment resulted in a considerable increase in the amount of time spent in the closed arms, as well as the frequency of entries into the closed arms, an effect that was not observed in control mice. In the ligand-treated group, the length of time and the number of entries into closed arms were reduced (Fig S4A). We next performed a pole test to monitor coordination. In the MPTP group, the overall time required by mice to reach the bottom of the pole and T-turn time were significantly prolonged to 18.1 and 4.40 s, respectively, compared with the control group, whereas in the ligand-treated group, they were shortened to 13.2 and 3.33 s, respectively (Fig 6B). Coordination was also monitored by a narrow beam walk test where mice injected with MPTP took longer time to traverse the beam with enhanced foot slip errors, which were reverted in the ligand-treated group (Fig S4B). We next performed a rotarod test where the foot movement of the mice was observed at different speeds. Compared with the poor performance of the MPTP group, the ligand-treated group had a longer time to fall off the rotating bar showing improved performance at all speeds (Fig 6C). To assess the neuromuscular strength, a wire hanging test was performed. While performing this test, animals were directed to grab the wire with their paws for 180 s or until they fell. It was observed that the ligand-treated group displayed better grip strength as compared to the MPTP group as evidenced by more latency to fall (Fig 6D).

Furthermore, to examine the effect of GW4064 and T3 on cognitive impairment caused by MPTP, we performed the Morris water maze test. The escape latency was recorded as an index of spatial learning. The time taken by MPTP-injected mice to get to the hidden platform was comparatively longer than the control group. However, the beneficial effect of GW4064 and T3 on learning was noticed during the 5-d experimental period (Fig 6E). In the probe trial experiment, impaired memory deficits were observed in the MPTP group, spending much less time in the target quadrant and shorter platform crossing duration compared with the control group. The ligand-treated group reached levels of performance equal to those of the controls suggesting their ability to memorize the platform position (Fig 6F).

### GW4064 and T3 effectively reduced MPP^+^-induced ER stress and ROS production in differentiated human neuronal SH-SY5Y cells

**Figure 7.**
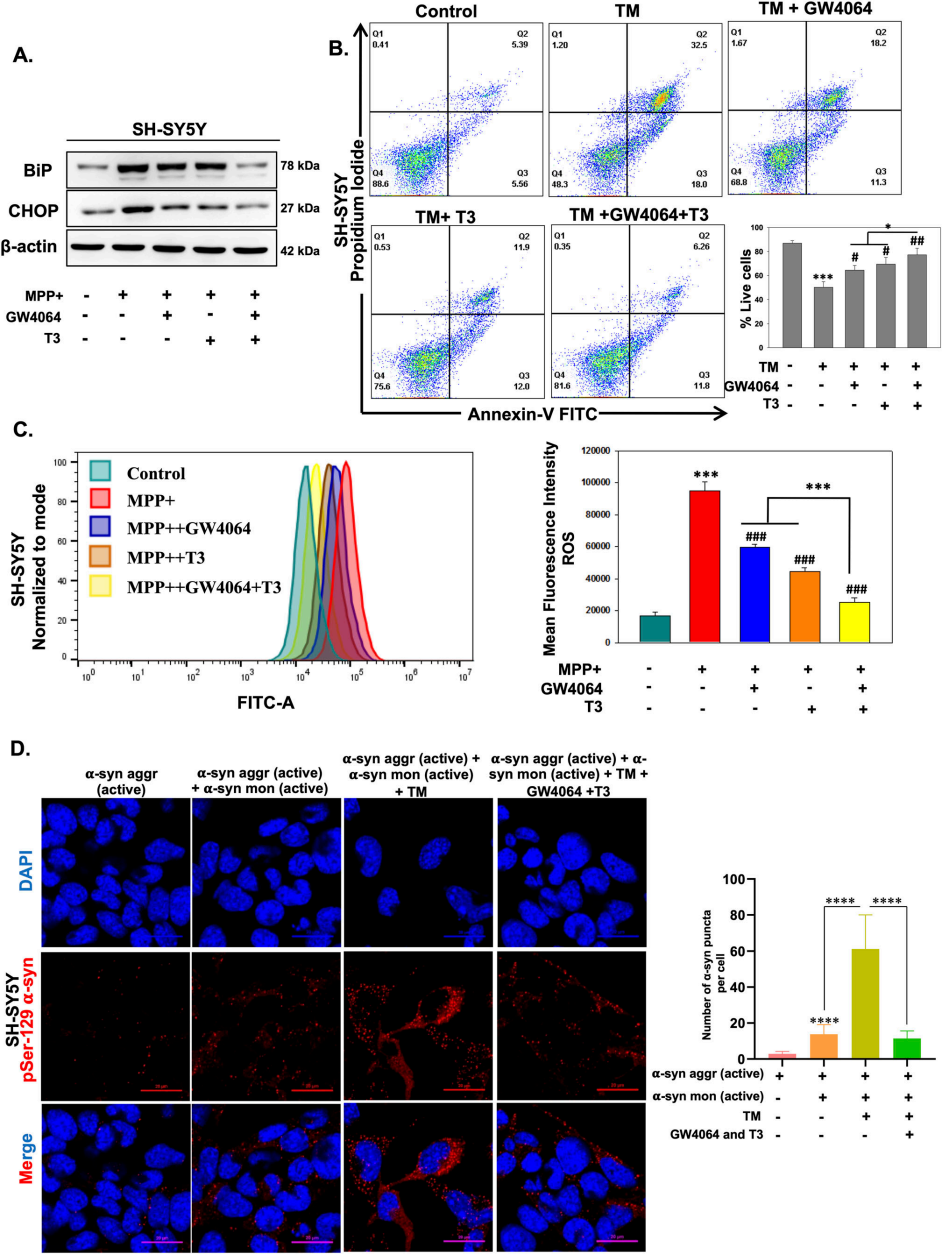
Activation of Nr1h4 and Thrb prevents ER stress and ROS production in human neuronal SH-SY5Y cells. SH-SY5Y cells were treated with MPP^+^ in the absence or presence of GW4064 or T3 or both for 24 h. **(A)** Immunoblot of ER stress markers BiP and CHOP. **(B)** Annexin-V and PI staining by flow cytometry. **(C)** ROS levels detected by flow cytometry. **(D)** Effect of ER stress and GW4064 and T3 on α-syn aggregation: (D) representative confocal images of 4 μg/ml α-syn aggregates (active) alone or in combination with active monomers, in the presence of TM (2.5 μg/ml) only or together with GW4064 and T3 for 24 h. The number of α-syn puncta per cell was counted for 30 cells in each group. Data are the mean ± SD or representative of three independent experiments performed in triplicates. *P < 0.05, **P < 0.01, ***P < 0.001, compared with control or as indicated; #*P* < 0.05, ##*P* < 0.01, ###*P* < 0.001, compared with MPP^+^ by a two-tailed *t* test.

**Figure S5.**
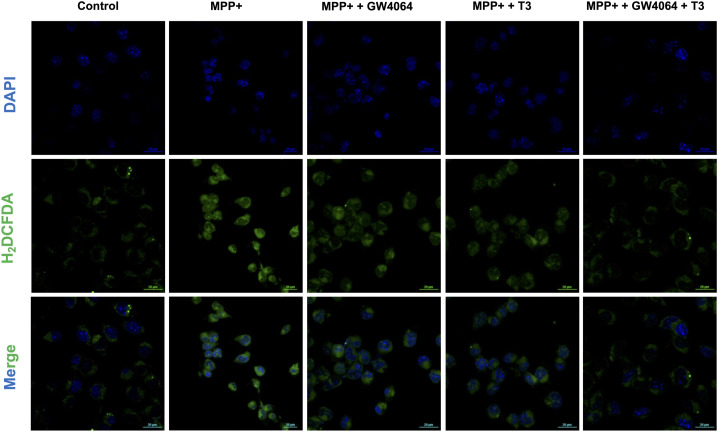
GW4064 and T3 treatment reduced MPP^+^-induced ROS production in SH-SY5Y cells. Total ROS levels were detected by confocal imaging. Data are representative of three independent experiments performed in triplicates.

**Figure S6.**
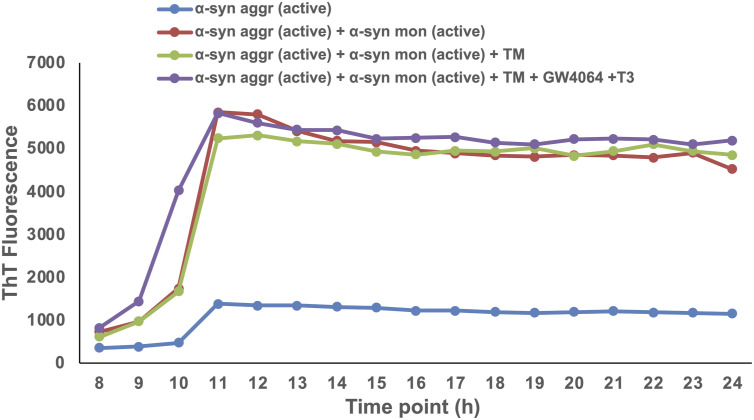
Dynamics of α-synuclein aggregation in the presence of TM and GW4064 and T3. ThT (25 μM) fluorescence binding assay of α-syn aggregates (active), in combination with α-syn monomer (active) and the presence of TM alone or together with ligands (GW4064 and T3).

We next monitored the effect of MPP^+^-induced ER stress in differentiated human neuronal SH-SY5Y cells. Treatment with GW4064 and T3 reduced ER stress, which was observed in MPP^+^-treated cells. Levels of BiP and CHOP were significantly reduced in combination ligand treatment signifying the relevance of activation of Nr1h4 and Thrb in human neuronal cells (Fig 7A). We next examined the TM-induced apoptosis by flow cytometry, and as expected, there was increased apoptosis in the differentiated SH-SY5Y cells after TM treatment, whereas ligand treatment prevented TM-induced cell death (Fig 7B). Next, to examine whether GW4064 and T3 suppressed MPP^+^-induced ROS production, we evaluated ROS generation in differentiated SH-SY5Y by both flow cytometry (Fig 7C) and confocal analysis (Fig S5). It was observed that ROS production in MPP^+^-treated cells was significantly reduced in both GW4064- and T3-treated cells. This reduction was more pronounced in combination with ligand treatment indicating the protective effects of activation of Nr1h4 and Thrb in human neuronal cells. Several studies have demonstrated that α-syn aggregate formation can elicit ER stress response. Elevated ER stress, in turn, promotes α-syn aggregation (Cóppola-Segovia et al, 2017). To investigate the impact of GW4064 and T3 on α-syn aggregation, cells were subjected to α-syn aggregates, active proteins existing in preformed fibrils. In addition, cells were treated with pre-incubated α-syn aggregates and active monomers. The α-syn aggregates serve as templates, or “seeds,” by recruiting active monomers into fibrils. Furthermore, treatment of TM, an ER stress inducer, leads to the increased punctation of phosphorylated α-syn aggregates. This pathology was decreased in cells treated with GW4064 and T3. These results suggested that the ligands are ameliorating TM-induced α-syn aggregate formation in differentiated SH-SY5Y cells (Fig 7D). To assess whether the ligands exert a direct effect on aggregation or act through ER stress pathways, we conducted a thioflavin-T (ThT) assay in a cell-free state. There was an increase in the fluorescence intensity when active α-syn aggregates were incubated with active monomers as compared to active α-syn aggregates alone. Interestingly, the fluorescence intensity of aggregates remained constant upon treatment with TM alone or in the presence of ligands (T3 and GW4064). This indicates that ligands did not have a direct effect on the aggregates (Fig S6). Ligands are able to reduce the aggregation in the cellular milieu by reducing the ER stress. These results substantiate the neuroprotective effects of GW4064 and T3 in human neuronal cells.

## Discussion

Protein synthesis and folding majorly take place in the ER, accounting for a substantial portion of a mammalian cell’s overall protein production. The transport of proteins through the ER must be scrutinized for any anomalies, such as the aggregation of misfolded and unfolded proteins, as a buildup of such proteins inside the ER could lead to a condition called ER stress. To respond to the accumulation of misfolded proteins within the ER, mammalian cells have evolved a complex network of signaling pathways collectively known as UPR. It aids in reestablishing cellular homeostasis after ER stress, but aberrant or sustained ER stress plays a potential role in the pathophysiology of a variety of diseases (Lin et al, 2008). This study sought to comprehensively address the implication of the differential expression of NRs on the outcome of ER stress. We used common ER stress inducers for the induction of ER stress in mouse neuronal cells (Fig S1). A thorough examination of all the NRs revealed the differential expressions of some of the NRs, which were either uniquely or commonly regulated in ER stress induced by TM, TG, and BFA (Fig 1A–C). Among these NRs, Nr1h4, Pparg, and Thrb were significantly up-regulated in all three stress conditions at both mRNA and protein levels (Fig 1D and E). PPARγ has previously been shown to alleviate ER stress in neuronal cells. It protects the brain against cerebral ischemia/reperfusion injury by reducing ER stress as evidenced by PPARγ-deficient cells, which had more severe neuron deficits and higher amounts of CHOP, cleaved caspase-12, and BiP (Chen et al, 2019). Nr1h4 is known to have a role in several metabolic processes in the liver, and it has been shown to curtail ER stress in liver injury and kidney disease (Gai et al, 2017; Han et al, 2018). Furthermore, bile acids such as TUDCA, acting as ligands for Nr1h4, have demonstrated neuroprotective potential in experimental animal models of various neurodegenerative conditions (Nunes et al, 2012; Bazzari et al, 2019; Xing et al, 2023). The suppression of ER stress represents a conceivable mechanism that governs the therapeutic potential of bile acids in addressing neurodegenerative diseases (Zhang et al, 2018; Dong et al, 2020). There are recent reports of the presence of functional Nr1h4 in brain regions (Huang et al, 2016). GW4064 has been shown to have beneficial effects in liver disease by reversing bile acid dysmetabolism, attenuating hepatic inflammation, and mitigating hepatotoxicity (Liu et al, 2018; Cao et al, 2019). T3 has also demonstrated efficacy in various disease contexts including traumatic brain injury, hyperoxia-induced lung injury, and numerous metabolic disorders (Crupi et al, 2013; Zhang et al, 2021). With no known reports of the functional relevance of Nr1h4 and Thrb in ER stress in neurons, we chose to decipher the outcome of gain and loss of function of these two receptors on ER stress. It was observed that activation of Nr1h4 and Thrb by their respective ligands ameliorates ER stress by modulating different UPR pathways (Fig 2A, B, D, and E). We next used the combination of ligands and found that it was more efficacious in attenuating TM-induced ER stress (Fig 2C and F). Our data suggest that Nr1h4 modulates PERK and ATF6 pathways, whereas Thrb regulates PERK and IRE1 pathways of UPR, resulting in a synergistic effect. Deteriorated cellular functions resulting from irreversible ER stress often lead to cell death (Sano & Reed, 2013). Multiple assays were employed to monitor ER stress–induced apoptosis, including immunoblot analysis of various pro- and anti-apoptotic markers and the activation of caspase-3 by a fluorescent assay. The results suggested that the combination of ligands was more effective in reducing ER stress–induced apoptosis (Fig 3). ER stress has implications in several diseases. In this study, we have studied ER stress in the physiology of PD where MPTP was used to mimic PD conditions both in vitro and in vivo. At cellular levels, MPTP is known to induce ER stress, mitochondrial dysfunction, and ROS production resulting in neuronal cell death (loss of dopaminergic neurons). We observed that the combination of GW4064 and T3 was more potent in mitigating ER stress as evidenced by decreased levels of ER stress markers. In addition, it maintained mitochondrial activity as demonstrated by recovered dissipated mitochondrial membrane potential and reduced ROS production measured by both flow cytometry (Fig 4) and confocal imaging (Fig S3). To support our in vitro findings, the MPTP mouse model of PD was generated (Fig 5A). This model offers several advantages, including its systemic administration, induction of non-motor symptoms, and the significant loss of over half of all dopaminergic neurons in the SNpc (Mustapha & Mat Taib, 2021). Although MPTP can elevate both total and phosphorylated α-syn levels in the SNpc, it seldom induces the formation of Lewy bodies (Hu et al, 2020). Genetic models can be valuable for studying specific aspects of PD. For instance, models involving the overexpression or mutation of α-syn can help us to better understand the formation of Lewy bodies (Dawson et al, 2010). However, the focus of this study was to investigate the mechanistic pathways underlying PD. The toxicity induced by MPTP correlates with ER stress, subsequently triggering mitochondrial dysfunction and the generation of ROS, and ultimately resulting in dopaminergic cell death (Meredith & Rademacher, 2011). These characteristics render the MPTP mouse model of PD particularly appropriate for the focus of this study. Data obtained from in vivo studies have led to a compelling conclusion that GW4064 and T3 can rescue animals from MPTP-induced ER stress and loss of TH-positive dopaminergic neurons in the striatum and the SNpc (Fig 5B and C). Impaired mobility is a characteristic feature of people with progressive nervous system disorders such as PD. Several behavioral tests were carried out to monitor the movement of mice after MPTP intoxication. These tests provide a very powerful tool to analyze the protective effects of small molecules in PD pathogenesis. Mobility was determined by the OFT, pole test, rotarod test, wire hanging test, and narrow beam walk test (Figs 6A–D and S4B) where ligand-treated animals performed better than MPTP-treated animals in all the tests performed. Animals receiving MPTP have been shown to exhibit anxiety as well, and we observed that MPTP-treated animals were more anxious extending the duration of time in closed arms, which was not the case for the ligand-treated group (Fig S4A). Non-motor features, such as cognitive decline, accompany other motor symptoms, and the Morris water maze test is an excellent experiment to monitor spatial learning and memory. Ligand-treated animals were able to reach the platform in less time as compared to MPTP-treated ones, and also, they were more potent in memorizing the platform position (Fig 6E and F). These results indicated that ligand treatment alleviated the severity of PD in the MPTP mouse model of PD. Protective effects of both the ligands were also observed in differentiated human neuronal cell line SH-SY5Y where GW4064 and T3 were able to reduce ER stress, apoptosis, and ROS production (Figs 7 and S5). Moreover, the pathophysiology of PD is profoundly affected by α-syn aggregation, which has been linked to several pathogenic processes such as ER stress and mitochondrial dysfunction. Given the significance of α-syn in PD, we observed that TM-induced α-syn aggregate formation was mitigated upon treatment with GW4064 and T3 (Fig 7D). The presence of misfolded proteins, such as α-syn, triggers the activation of adaptive UPR signaling (Colla, 2019). This response works to alleviate ER stress, restore ER homeostasis, and prevent the worsening of PD pathogenesis, indicating neuron’s ability to handle mild or basic ER stress. Persistent ER stress and excessive UPR activation can, however, result in neuronal cell death. Therefore, in the advanced stages of PD when maladaptive UPR signals dominate, it becomes crucial to suppress UPR signaling to prevent neuronal apoptosis. In addition to suppressing the UPR, it is imperative that α-syn undergoes degradation through clearance mechanisms such as the ubiquitin/proteasome pathway or autophagy.

In summation, this work is the first one to discuss the importance of Nr1h4 and Thrb in alleviating ER stress and associated cell death in neuronal cells. In this study, we have demonstrated for the first time that GW4064 and T3 exhibit protective effects in the MPTP mouse model of PD by alleviating ER stress. We used concentrations that are minimal yet effective in activating their corresponding receptors to avoid toxicity and any other potential side effects. Moreover, the synergistic action of these ligands in alleviating ER stress by virtue of targeting different arms of UPR suggests that their combined use may offer enhanced therapeutic value and hold potential for mitigating certain aspects of PD pathology. Although this study provided a platform to consider these molecules in ER stress research and associated diseases, further studies are needed to translate their beneficial effects into therapeutics. It would be interesting to also look for the effects of these ligands on other physiologies of PD such as excitotoxicity and neuroinflammation as well.

## Materials and Methods

### Animals & ethics statement

C57BL/6 male mice (6–8 wk old) were obtained from the Jackson Laboratory and were kept in a disease-free environment with adequate food and water in the institute’s animal house facility. Mouse experiments were approved by the Institutional Animal Ethics Committee and were conducted in accordance with the National Regulatory Guidelines stated by the Committee for the Purpose of Supervision of Experiments on Animals (No.55/1999/CPCSEA), Ministry of Environment and Forest, Government of India.

### Cell culture and reagents

Mouse N2a cells and human SH-SY5Y (ATCC) were grown in DMEM and DMEM/F12 media, respectively, supplemented with 100 U/ml penicillin–streptomycin and 10% FBS, and maintained at 37°C and 5% CO_2_. For differentiation of N2a into dopaminergic neurons, media were changed to DMEM+ 0.5% FBS with 1 mM dbcAMP for 24–48 h (Tremblay et al, 2010). To differentiate SH-SY5Y cells, 10 μM retinoic acid was added for 3 d, and after 3 d, media were changed and fresh media containing 16 nM phorbol 12-myristate 13-acetate were added for another 3 d. For every experiment in N2a and SH-SY5Y cells, 5 μM GW4064 and 10 nM T3 were used.

### PCR array

RNA was isolated from cells using RNeasy Mini Kit (QIAGEN). cDNA was prepared from 1 μg of total RNA using RT^2^ First Strand Kit (QIAGEN) followed by qRT–PCR using RT^2^ SYBR Green ROX qRT-PCR Mastermix (QIAGEN). Customized PCR array plates (QIAGEN) were used to ascertain the expression profile of all the murine NRs and associated co-regulators as per the manufacturer’s instructions. Relative fold regulation was determined by the 2^−ΔΔCt^ method. The NRs with a Ct value less than 35 were only included in the analysis (Saini et al, 2018).

### Measurement of mitochondrial membrane potential (Δψm)

A membrane-permeant JC-1 dye was used to measure *Δψm* in MPP^+^-treated differentiated N2a cells using flow cytometry analysis as per the manufacturer’s instructions. Briefly, cells at a density of 5 × 10^5^ cells per well were seeded in a 12-well plate and were treated with GW4064 or T3 or both for 3 h followed by MPP^+^ treatment for 24 h. The cells were then suspended in 1 ml PBS. For a positive control, carbonyl cyanide m-chlorophenyl hydrazone at 50 μM concentration was used. Cells were treated with 2 μM JC-1 and kept for 30 min at 37°C and 5% CO_2_, cells were then washed with PBS twice, and the pellet was resuspended in 300 μl PBS. Samples were acquired on a flow cytometer where standard compensation was performed using a carbonyl cyanide m-chlorophenyl hydrazone–treated sample. The lower red (JC-1 aggregates)/green (JC-1 monomers) fluorescence intensity ratio is suggestive of mitochondrial depolarization (Sivandzade et al, 2019).

### Cell viability assay

The cell viability was assessed using an MTT dye. Differentiated N2a cells at a density of 5 × 10^3^ cells/well were seeded and pretreated with either GW4064 or T3 or both for 3 h and then incubated with or without TM for 24 h. Cells were then stained with an MTT dye (5 mg/ml, 37°C, 4 h), and a solubilization solution was added to dissolve the formazan crystals. Absorbance at 570 nm was measured using a microplate reader. The cell cytotoxicity assay was performed using BioVision’s LDH Cytotoxicity Assay Kit II (#K313-500) as per the manufacturer’s protocol. Briefly, cells were seeded in a 96-well plate and the reaction was performed in triplicate. 100 μl culture medium with no cells was taken as the background control, 100 μl cells only were taken as low control, 100 μl cells with 10 μl cell lysis solution added were taken as high control, and 100 μl cells with TM alone or TM along with GW4064 or T3 or both were taken as test samples. 10 μl of each sample medium was transferred to a 96-well plate, and 100 μl LDH reaction mix was added to each well, mixed, and incubated for 30 min at room temperature. The absorbance of all the controls and samples was measured with a plate reader at 450 nm, with a reference wavelength of 650 nm. The % cytotoxicity was determined using the following formula:

Cytotoxicity (%)=(Test sample−Low control)/(High control−Low control)×100

### Measurement of ROS production

Briefly, 5 × 10^5^ cells/well were plated for 24 h in a 12-well plate with or without coverslips followed by treatment with GW4064 or T3 or both for 3 h, and later incubated with MPP^+^ for 24 h. Intracellular ROS was measured by labeling cells with 2.5 μM of H_2_DCF-DA (D399; Molecular Probes) for 30 min at 37°C. Fluorescence intensity was monitored by flow cytometer using 488-nm lasers and by confocal microscopy.

### Caspase-3 assay

Activity of caspase-3, a critical executioner of apoptosis, was detected by Caspase-3 Activity Assay Kit (#5723; CST) according to the manufacturer’s instructions. It is a fluorescent assay that contains a fluorogenic substrate for caspase-3 where activated caspase-3 cleaves this substrate generating high fluorescence. After treatment according to the experimental design, the cell lysate was prepared, and 40 μg/ml lysate was diluted in assay buffer. 200 μl of substrate solution B and 25 μl of lysate solution were mixed in a 96-well black plate followed by incubation at 37°C in dark for 2–4 h. Relative fluorescent units were determined with excitation at 380 nm and emission at 420–460 nm on a fluorescence plate reader.

### Annexin-V and PI staining

Briefly, 5 × 10^5^ cells per well were plated onto 12-well plates and treatments were given as mentioned in the Results section. Cells were harvested after 24 h, washed with cold 1X PBS twice, and stained with annexin-V and PI using BD Biosciences Kit (#556547) according to the manufacturer’s instructions. Cells were resuspended in 100 μl 1X binding buffer with 5 μl annexin-V and PI followed by 15-min incubation in dark at RT. The cells were acquired on a BD Accuri C6 flow cytometer, and data were analyzed by FlowJo software (Bhagyaraj et al, 2019). Apoptotic cells comprising both late apoptotic (annexin-V–positive and PI-positive) and early apoptotic (annexin-V–positive and PI-negative) cells were quantified.

### RNA isolation and quantitative real-time PCR (qRT–PCR)

Using TRIzol reagent, total RNA was extracted from the cells and cDNA was prepared from 1 μg of total RNA using a verso cDNA synthesis kit. The expression of various ER stress genes was determined by *qRT–PCR* using gene-specific primers and DyNAmo ColorFlash SYBR Green qPCR Kit. For normalization, β-actin was used as a housekeeping gene. The 2^−ΔΔCt^ method was used to calculate the fold change.

### PD animal model

C57BL/6 male mice (8 wk old) were procured from the animal facility at CSIR-IMTECH, and all experiments received approval from the Institutional Animal Ethics Committee of IMTECH as stated earlier. Mice were categorized into three groups (15 mice per group): Group I, normal control, received saline; Group II, received MPTP (30 mg/kg, i. p.); and Group III, received MPTP treatment (30 mg/kg, i. p.) + GW4064 (30 mg/kg, i. p.) and T3 (250 μg/kg, i. p.). The MPTP-treated groups received five injections of MPTP-HCl (30 mg/kg, i. p.) in saline every day for a consecutive 5 d. Group III received ligand treatment 4 h before MPTP administration. 2 d after the last injection, behavioral assessments were performed, then all the animals were euthanized, and brains were isolated for further analysis (Meredith & Rademacher, 2011).

### siRNA knockdown

For transient silencing of Nr1h4 and Thrb in differentiated N2a cells, a final dose of 60 nM of either a scrambled control siRNA or gene-specific siRNA was used to transfect cells at 70% confluency using TurboFect Transfection Reagent for 48 h according to the manufacturer’s instructions.

### Western blot analysis

Immunoblot analysis was performed on differentiated N2a and SH-SY5Y cell extracts, and brain extracts of experimental mice. Protein concentrations in the samples were assessed using Bradford’s reagent. SDS–PAGE was used to separate equal amounts of proteins, which were then transferred to a polyvinylidene difluoride membrane (Immobilon-P, IPVH00010; Millipore) for 2 h. The membrane was blocked with blocking buffer (5% BSA in Tris-buffered saline/Tween-20) for 2 h at RT. Blots were then probed with primary antibodies overnight at 4°C. After washing (three times for 5 min each with Tris-buffered saline/Tween-20 buffer), the membrane was then probed with a secondary antibody conjugated with horseradish peroxidase and visualized by chemiluminescent HRP substrate Luminata Forte (WBLUF0500; Millipore). Primary antibodies used were as follows: BiP (C50B12) (#3177; CST), CHOP (L63F7) (#2895; CST), PERK (#5683 D11A8; CST), phospho-PERK (Thr980) (16F8) (#3179; CST), eIF2α (#9722; CST), phospho-eIF2α (Ser51) (#9721; CST), ATF-4 (D4B8) (#11815; CST), ATF6 (70B1413.1) (#NBP1-40256SS; Novus Biologicals), IRE1α (14C10) (#3294; CST), phospho-IRE1α (pSer724) (#NB100-2323SS; Novus Biologicals), XBP1 (#NBP1-77681SS; Novus Biologicals), tyrosine hydroxylase (E2L6M) (#58844; CST), Bax (D2E11) (#5023; CST), cleaved PARP1 (E51) (#ab32064; Abcam), Mcl-1 (D35A5) (#5453; CST), Bcl-2 (50E3; CST), farnesoid X receptor (D-3) (#sc-25309; SCBT), Thrb (#ab5622; Abcam), PPARγ (E−8) (#sc-7273; SCBT), β-actin (C4) (#sc-47778; SCBT).

### IHC

Brains were removed carefully from three mice per group and were dehydrated with a graded series of ethanol after being fixed in 4% PFA for 24 h. They were then embedded in paraffin blocks, and 4-μm-thick sections were mounted on the slide followed by staining with anti-TH antibody.

### Brain sections and tissue preparation

After performing behavioral tests, three mice per group were used for the preparation of brain sections for IHC. The brains were removed from the remaining mice and quickly frozen in liquid nitrogen. The two striata from each hemisphere were isolated and used for Western blot analysis.

### Confocal imaging

Differentiated SH-SY5Y cells were treated with human α-syn protein aggregates (active) (ab218819, 4 μg/ml; Abcam) and human α-syn protein monomer (active) (ab218818; Abcam). Immunostainings were performed as described previously. Differentiated SH-SY5Y cells were fixed with 4% PFA and subsequently incubated with primary antibodies against phosphorylated S129 α-syn (α-syn-P; 1:1,000; ab184674) at 4°C for 24 h. After washing 3× with PBS, the cells were incubated with Alexa Fluor 647 anti-mouse secondary antibody (1/500) for 1 h at RT, counterstained with Hoechst (blue) nuclear stain (1/4,000), and mounted on slides with antifade reagent. Images were acquired by a Nikon AIR confocal microscope using a 60× objective.

### ThT assay

A ThT assay was performed as described previously (Xue et al, 2017). Following the manufacturer’s protocol, a 25 μM ThT solution was prepared in PBS and incubated with human α-syn protein aggregates (active) (10 μM) and monomers (active) (100 μM) at a 1:10 ratio in a 96-well black plate. Subsequently, the mixture of active α-syn aggregates with monomers and ThT was incubated with TM in the presence or absence of T3 (10 nM) and GW4064 (5 μM). Fluorescence measurements were taken at 450-nm excitation and 485-nm emission using a plate reader set at 37°C with linear shaking at 30-s intervals.

### Behavioral tests

#### OFT

OFT was conducted to assess the exploratory and anxiety-linked behavior of mice, where activity was limited in the closed box (50 × 50 × 50 cm) with a white smooth floor. Mice were gently placed in the center of the open field arena to allow for their free movement for 15 min, and a webcam positioned above the arena was used to record the activity of mice using computer tracking software ANY-maze. The arena was separated into two zones by tracking software: the center zone and the corner zone. Several parameters such as the overall distance traveled, the number of entries, and the amount of time spent in the center zone were measured for each mouse (Seibenhener & Wooten, 2015).

### Narrow beam walk test

To determine the motor coordination requiring stability and balance, a narrow beam walk test was performed. Training was provided to all the animals to move on a narrow, flat immovable wooden beam (L100 cm × W1 cm) that was 50 cm above the ground and fitted with a covered escape platform (20 × 20 × 20 cm) at one end of the beam. The time taken to traverse from one end of the beam to the other and the number of foot slip errors were measured as reported earlier (Luong et al, 2011).

### Wire hanging test

The wire hanging test is used to assess muscle strength and coordination, which is impaired in the mouse PD model. Mice were trained to grasp a horizontal metallic wire (55 cm long) placed 38 cm above the home cage and were gently turned upside-down along the axis of the wire. Three trials for 3 min with 30-s intervals were performed, and latency to fall was recorded (Hutter-Saunders et al, 2012).

### EPMT

The comprehensive assessment of anxiety-related behavior can be provided by EPMT by examining multiple parameters. The EPM device consisted of four arms (30 × 5 cm): two opposite open arms and two opposite closed arms equipped with high black walls (15 cm), elevated to a height of 45 cm above the floor. Each mouse was kept at the central square facing one of the two open arms of the maze. Mice’s behavior was captured for 15 min. The duration and the number of entries into the open and closed arms were noted using ANY-maze software. The tendency of mice to avoid entry into the open arms is noted as an index of anxiety (Lister, 1987).

### Pole test

A pole test was performed to elucidate the movement disorders. Animals were placed on a vertical iron pole (1 cm in diameter and 60 cm high) grasping the pole with their four paws and their head upward. The pole’s base was positioned inside a mouse home cage with bedding material. The time required to orient 180° downward (“T-turn”) and the time taken to reach the bottom of the pole back into the cage were recorded. The maximum time allowed was 60 s (Matsuura et al, 1997).

### Rotarod test

To examine grip strength and motor coordination, a rotarod test (Orchid Scientific) was performed. Mice were trained for three consecutive days for 5 min each. Mice received five trials for which the rod was accelerated from 5 to 20 rpm over the time of 3 min during the training period. The intertrial interval was 5 min. On the experimental day, latency to fall from the rotating rod for each mouse at four different rotation speeds was recorded (Rozas et al, 1998).

### Morris water maze test

The spatial learning and memory were determined by the Morris water maze test. The apparatus includes a black circular pool (40 cm in height and 120 cm in diameter), filled with water to a depth of 20 cm. The movement of mice was tracked using a camera linked to ANY-maze video tracking software. Four equally sized quadrants were created in the pool. To make the water opaque and background light, milk was added to water. The test was conducted in two phases. The first phase consisted of hidden platform training where mice were trained for five consecutive days to find a hidden platform placed 1 cm below the water surface in one of the four quadrants. Mice were gently placed in water facing the pool’s wall, and the amount of time it took to find the hidden platform was measured as escape latency (a measure of spatial learning). The maximum time was 90 s. Whenever a mouse took longer than 90 s to reach the platform, the experimenter would lead the mouse there and its escape latency was recorded as 90 s. After reaching the platform, each mouse was permitted to stay on the platform for 30 s. At the end, animal was dried and placed back in its cage. Memory was assessed in the second phase of the experiment, which consisted of a spatial probe trial that was performed on the sixth day after the 5-d training phase. The platform was taken out of the water, and mice were positioned in the quadrant diagonally opposite to the platform location with a cutoff time of 60 s. The duration of the time in the target quadrant (quadrant where the platform was positioned during training) and the number of times animals crossed the original platform location were recorded (Terry, 2009).

### Statistical analysis

The statistical analysis was performed using SigmaPlot or GraphPad Prism software. The normality of the data was assessed using the D'Agostino–Pearson omnibus test. Results are presented as the mean ± SD or SEM. Statistical analysis was performed using a two-tailed *t* test for comparisons between two groups. To evaluate the differences between more than two groups, one-way analysis of variance (ANOVA) was employed, followed by either the Tukey post hoc analysis (in cases of homogeneous variance) or Dunnett’s T3 test (for heterogeneous variance). Significance levels are reported with symbols in the figures (*comparisons with control or as indicated and ^#^comparisons with TM) where the following thresholds for statistical significance were set: *, #*P* < 0.05; **, ##*P* < 0.01; and ***, ###*P* < 0.001.

### The paper explained the following:

#### Problem

ER stress is one of the potential molecular mechanisms driving neurodegenerative diseases such as Parkinson’s disease. Therapeutic drugs targeting ER stress might serve as an effective treatment strategy for such diseases.

#### Results

Activation of Nr1h4 and Thrb by their ligands, GW4064 and T3, respectively, ameliorated ER stress by modulating different UPR pathways. The combination of GW4064 and T3 showed promising effects in reducing ER stress, maintaining mitochondrial activity, and protecting dopaminergic neurons in a PD model. Ligand-treated animals exhibited improved mobility, reduced anxiety, and enhanced cognitive function compared with MPTP-treated mice.

#### Impact

The study highlighted the potential of GW4064 and T3 as therapeutic ligands for PD and their role in alleviating ER stress and cell death in neuronal cells.

## Supplementary Material

Reviewer comments

## Data Availability

The data that support the findings of this study will be made available to researchers on reasonable request. The data are also submitted to CSIR-IMTECH, Sector 39A, Chandigarh, Institutional Repository. Mouse experiments were approved by the Institutional Animal Ethics Committee and were conducted in accordance with the National Regulatory Guidelines stated by the Committee for the Purpose of Supervision of Experiments on Animals (No.55/1999/CPCSEA), Ministry of Environment and Forest, Government of India.
